# Crucian Carp-Derived ACE-Inhibitory Peptides with In Vivo Antihypertensive Activity: Insights into Bioactivity, Mechanism, and Safety

**DOI:** 10.3390/molecules30132812

**Published:** 2025-06-30

**Authors:** Runxi Han, Jingshan Tian, Yingge Han, Guoxiang Wang, Guanghong Zhou, Chen Dai, Chong Wang

**Affiliations:** 1 College of Food Science and Technology, Nanjing Agricultural University, Nanjing 210095, China; 9221810430@stu.njau.edu.cn; 2 College of Life Sciences, Nanjing Agricultural University, Nanjing 210095, China; 10123104@stu.njau.edu.cn (J.T.); 9241010329@stu.njau.edu.cn (Y.H.); 3 Experimental Teaching Center of Life Science, College of Life Sciences, Nanjing Agricultural University, Nanjing 210095, China; wanggx@njau.edu.cn; 4 State Key Laboratory of Meat Quality Control and Cultured Meat Development; Jiangsu Collaborative Innovation Center of Meat Production and Processing, Quality and Safety Control, College of Food Science and Technology, Nanjing Agricultural University, Nanjing 210095, China; ghzhou@njau.edu.cn; 5 Sanya Institute of Nanjing Agricultural University, Sanya 572024, China

**Keywords:** angiotensin-I-converting enzyme, bioactive peptide, crucian carp swim bladder, hypertension, molecular docking

## Abstract

This study explores the identification, characterization, and biological evaluation of angiotensin I-converting enzyme (ACE)-inhibitory peptides derived from enzymatic hydrolysates of crucian carp swim bladders. Following sequential purification by size-exclusion and reversed-phase chromatography, two bioactive peptides—Hyp-Gly-Ala-Arg (Hyp-GAR) and Gly-Ala-Hyp-Gly-Ala-Arg (GA-Hyp-GAR)—were identified using ultra-high-performance liquid chromatography coupled with linear ion trap–Orbitrap tandem mass spectrometry. The synthetic peptides demonstrated potent ACE-inhibitory activity in vitro, with IC₅₀ values of 12.2 μM (Hyp-GAR) and 4.00 μM (GA-Hyp-GAR). Molecular docking and enzyme kinetics confirmed competitive inhibition through key interactions with ACE active site residues and zinc coordination. In vivo antihypertensive activity was evaluated in spontaneously hypertensive rats, revealing that GA-Hyp-GAR significantly reduced systolic blood pressure in a dose-dependent manner. At a dose of 36 mg/kg, GA-Hyp-GAR reduced systolic blood pressure by 60 mmHg—an effect comparable in magnitude and timing to that of captopril. Mechanistically, GA-Hyp-GAR modulated levels of angiotensin II, bradykinin, endothelial nitric oxide synthase, and nitric oxide. A 90-day subchronic oral toxicity study in mice indicated no significant hematological, biochemical, or histopathological alterations, supporting the peptide’s safety profile. These findings suggest that GA-Hyp-GAR is a promising natural ACE inhibitor with potential application in functional foods or as a nutraceutical for hypertension management.

## 1. Introduction

Hypertension is a widespread, age-associated chronic condition and a major contributor to cardiovascular and cerebrovascular morbidity and mortality. Annually, complications from hypertension account for approximately 9.4 million deaths worldwide, and its prevalence continues to rise at an alarming rate [1]. Projections suggest that by 2025, nearly 156 million individuals globally could be affected, emphasizing the critical need for more effective management strategies [2].

Blood pressure regulation is closely linked to the renin–angiotensin system, with the angiotensin I-converting enzyme (ACE) playing a central role. ACE converts angiotensin I into angiotensin II (AngII), a potent vasoconstrictor that raises blood pressure, and simultaneously degrades bradykinin (BK), a vasodilator, thereby reducing its availability [3]. Consequently, synthetic ACE inhibitors such as captopril, lisinopril, and enalapril are widely employed in clinical settings to manage hypertension [4]. However, the prolonged use of these agents has been associated with several adverse effects, including electrolyte imbalances, renal impairment, dry cough, and angioedema. Specifically, hyperkalemia occurs in approximately 1–2% of patients, and renal impairment is more common in individuals with pre-existing renal conditions [5]. Dry cough and angioedema are also frequently reported side effects, particularly due to the accumulation of bradykinin, a known mediator of these symptoms [6,7]. These well-documented safety concerns have spurred the search for alternative antihypertensive therapies that offer both efficacy and a more favorable safety profile, particularly for long-term use.

In this context, food-derived bioactive peptides have emerged as promising candidates due to their natural origin and reported ACE-inhibitory effects [8]. For example, lactotripeptides from milk have been extensively documented for their ACE-inhibitory activity and resultant antihypertensive effects [9], while peptides extracted from wheat and soy proteins have demonstrated both blood-pressure-lowering and antioxidant properties, further supporting cardiovascular health [10]. Despite these encouraging findings, it is important to acknowledge that the therapeutic application of such peptides is still in its early stages. Several challenges—including poor gastrointestinal stability, limited bioavailability, short half-life in circulation, dose-dependent efficacy, and long-term safety concerns—currently hinder their translation into clinical use [11].

Building on these developments, recent research has increasingly focused on isolating ACE-inhibitory peptides from collagen, a major structural protein in animal connective tissues and a rich source of bioactive peptides [12,13]. For example, Cao et al. successfully identified a potent ACE-inhibitory peptide from bovine bone gelatin hydrolysate [14]. While terrestrial sources have traditionally dominated collagen research, fish-derived collagen is gaining attention because it has a lower risk of transmitting infectious diseases and fewer ethical concerns [15]. Despite its promise, research on fish-derived collagen peptides faces several challenges. First, the extraction process is more complex. Fish skin and scales, which are the most studied sources of collagen, contain significant amounts of glycosaminoglycans (e.g., chondroitin sulfate) and highly cross-linked collagen structures, requiring longer enzymatic or acidic hydrolysis processes for effective extraction [16]. Second, clinical studies on fish-derived collagen peptides are still in their early stages, and much work remains to establish their long-term safety, efficacy, and bioavailability. These challenges underscore the need for further research to optimize extraction methods and expand clinical trials to unlock the full potential of marine-derived collagen peptides.

Crucian carp (*Carassius auratus*) is one of the most commonly farmed freshwater fish species in China, with an annual production of approximately 2.772 million tons in 2018 [17]. Despite this large-scale output, crucian carp swim bladders are often discarded during processing due to their small size, leading to both the waste of valuable biomass and an increased environmental burden [18]. Notably, the swim bladder is rich in type I collagen, similar to other collagenous materials known to yield ACE-inhibitory peptides upon enzymatic hydrolysis. Compared to fish scales, swim bladders typically contain fewer heterologous proteins and glycosaminoglycans, making the extraction of high-purity collagen potentially simpler, requiring less enzymatic or chemical treatment [19].

While previous studies have demonstrated the anti-inflammatory and alcohol dehydrogenase-activating properties of crucian carp swim bladder hydrolysates, its potential as a source of ACE-inhibitory peptides remains unexplored [20,21]. Recognizing this gap, the present study aimed to investigate the crucian carp swim bladder as a sustainable and underutilized source of ACE-inhibitory peptides. The specific objectives were (1) to isolate and identify active peptides from enzymatic hydrolysates, (2) to elucidate their molecular interactions with ACE via docking and kinetic analysis, and (3) to evaluate their antihypertensive efficacy and long-term safety in vivo.

## 2. Results

### 2.1. Isolation and Purification of ACE-Inhibitory Peptide

**Figure 1:**
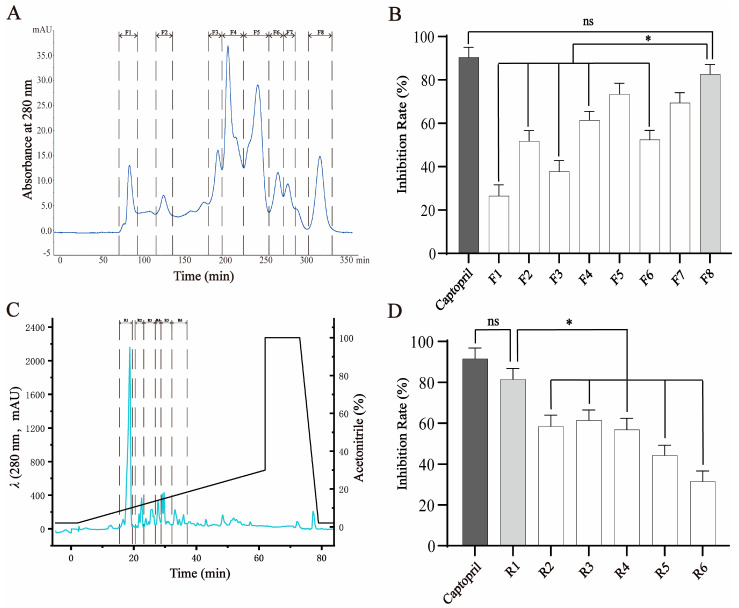
Fractionation and purification of ACE-inhibitory peptides from crucian carp swim bladder hydrolysates. (**A**) Gel filtration chromatography (Sephadex G-25) of the enzymatic hydrolysate yielded eight fractions (F1–F8), monitored at 280 nm. (**B**) ACE-inhibitory activities of fractions F1–F8. Fraction F8 exhibited the highest inhibitory activity among all fractions, though slightly lower than that of captopril (as positive control). (**C**) Reverse-phase HPLC separation of F8 using a linear acetonitrile gradient in 0.2% formic acid (3–63 min), resulting in six subfractions (R1–R6). (**D**) ACE-inhibitory activities of subfractions R1–R6. R1 exhibited the strongest activity and was selected for further purification and peptide identification. Data are expressed as mean ± SD (*n* = 3). * *p* < 0.05; ns = not significant.

Peptides hydrolyzed from crucian carp swim bladders were initially subjected to gel filtration chromatography using a Sephadex G-25 column, which separates components based on molecular size. As expected, the higher-molecular-weight peptides eluted earlier and eight distinct fractions (F1–F8) were collected (Figure 1A). These fractions were evaluated for their ability to inhibit ACE activity. Among them, F8, containing the lowest-molecular-weight peptides, exhibited the strongest ACE-inhibitory activity (81.98 ± 2.52% at 1 mg/mL), which was significantly greater than that of F1–F4 (*p* < 0.05), although slightly lower than that of the positive control, captopril (92.61 ± 2.07% at 10 μg/mL) (Figure 1B). The activity of F8 was considered comparable to that of captopril and therefore selected for further purification.

To further enrich bioactive components, F8 was subjected to RP-HPLC, which separates peptides based on hydrophobicity. Six subfractions (R1–R6) were collected according to their elution profile (Figure 1C). Among these, R1, the first eluting and least hydrophobic subfraction, showed the highest ACE-inhibitory activity (80.03 ± 2.43% at 1 mg/mL), significantly outperforming the other subfractions and showing no statistical difference compared to captopril (Figure 1D). Based on its strong activity, R1 was selected for subsequent peptide identification and structural characterization.

### 2.2. Identification and Validation of Peptides in R1 Fractions

**Figure 2:**
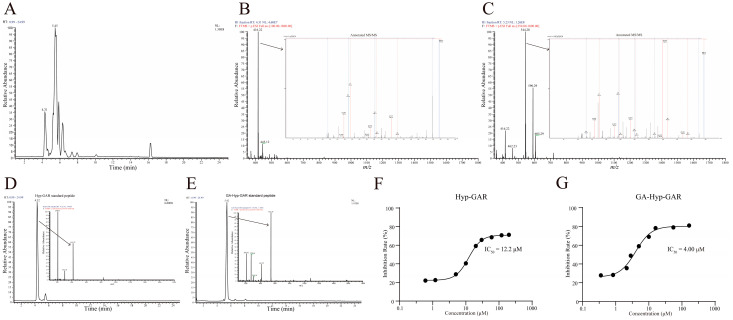
Structural identification and ACE-inhibitory activity of two bioactive peptides from crucian carp swim bladder hydrolysate. (**A**) Base peak chromatogram of the R1 subfraction analyzed by UHPLC-Orbitrap MS/MS. (**B**,**C**) MS/MS fragmentation spectra of the identified peptides Hyp-GAR and GA-Hyp-GAR, respectively. (**D**,**E**) Extracted ion chromatograms of synthetic Hyp-GAR and GA-Hyp-GAR, confirming identity based on matching retention times and fragment ion profiles. (**F**,**G**) Dose–response curves of Hyp-GAR and GA-Hyp-GAR showing their ACE-inhibitory activity. GA-Hyp-GAR exhibited a lower IC₅₀ value (4.00 μM) compared to Hyp-GAR (12.2 μM), indicating superior inhibitory potency.

Liquid-chromatography–tandem-mass-spectrometry (LC-MS/MS) is now a well-established and widely utilized method for accurate and high-throughput peptide sequencing. The base peak ion chromatogram of the R1 fraction, obtained via UHPLC-LTQ-Orbitrap analysis, is presented in Figure 2A. Detailed analysis focused on two prominent peaks with retention times of 4.31 min and 5.45 min, respectively. The peak at 4.31 min exhibited a molecular ion with an *m*/*z* value of 416.22 (Figure 2B). Peptide sequencing using PEAKS software (Version: XPro) identified this peak as the peptide Hyp-GAR, where “Hyp” denotes hydroxyproline. Similarly, the peak at 5.45 min had an *m*/*z* value of 544.28, corresponding to the peptide GA-Hyp-GAR (Figure 2C).

To verify the accuracy of the sequencing results, the synthetic peptides Hyp-GAR and GA-Hyp-GAR were analyzed under identical chromatographic and mass spectrometric conditions. As shown in Figure 2D,E, the retention times and *m*/*z* values of the synthetic peptides were in full agreement with those observed in the R1 fraction, thereby confirming the reliability of the peptide identification. Subsequently, the ACE-inhibitory activities of the synthetic peptides were assessed. As illustrated in Figure 2F,G, the IC₅₀ values for Hyp-GAR and GA-Hyp-GAR were determined to be 12.2 μM and 4.00 μM, respectively, indicating notable ACE-inhibitory activity.

### 2.3. Molecular Docking of Hyp-GAR and GA-Hyp-GAR

**Figure 3:**
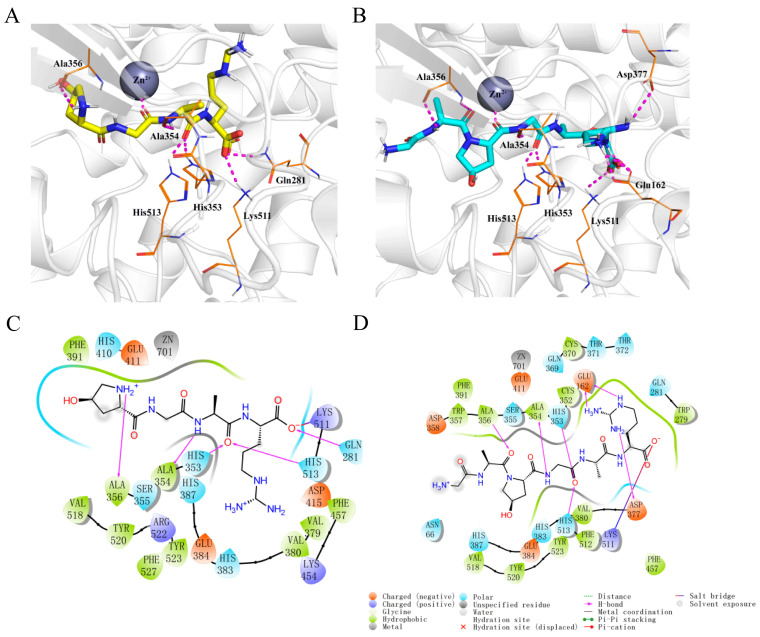
Molecular docking analysis of peptides Hyp-GAR and GA-Hyp-GAR with angiotensin-I-converting enzyme (ACE). (**A**,**B**) Three-dimensional docking models showing the binding conformations of Hyp-GAR (**A**) and GA-Hyp-GAR (**B**) within the active site of ACE. Key interacting residues are labeled, and the zinc ion (Zn^2^⁺) is represented as a grey sphere. (**C**,**D**) Two-dimensional interaction diagrams of Hyp-GAR (**C**) and GA-Hyp-GAR (**D**) with ACE, highlighting hydrogen bonds, salt bridges, hydrophobic interactions, and metal coordination.

Molecular docking simulations demonstrated that both Hyp-GAR and GA-Hyp-GAR adopt similar orientations within the active site of ACE. Both peptides formed stable complexes with ACE through a combination of hydrogen bonding, electrostatic interactions, and hydrophobic contacts (Figure 3A,B). As shown in Figure 3C, Hyp-GAR formed five hydrogen bonds and one salt bridge with key ACE residues, including Gln281, His353, Ala354, Ala356, Lys511, and His513. The calculated docking free energy for Hyp-GAR was −8.6 kcal/mol. In contrast, GA-Hyp-GAR engaged in seven hydrogen bonds and one salt bridge, interacting with residues Glu162, His353, Ala354, Ala356, Asp377, Lys511, and His513 (Figure 3D). The corresponding docking free energy was −9.5 kcal/mol, indicating a stronger binding affinity compared to Hyp-GAR. This enhanced binding energy supports the superior in vitro inhibitory potency of GA-Hyp-GAR, as reflected by its lower IC₅₀ value.

To validate the reliability of the docking protocol, two well-known ACE inhibitors—lisinopril and captopril—were also docked to the same ACE structure (PDB ID: 1O8A) under identical parameters. The predicted binding energies for lisinopril and captopril were −8.09 kcal/mol and −5.54 kcal/mol, respectively, which are less favorable than those observed for Hyp-GAR (−8.60 kcal/mol) and GA-Hyp-GAR (−9.50 kcal/mol). These results suggest that the identified peptides may exhibit comparable or stronger binding affinity toward ACE. Generally, a binding energy lower than −5 kcal/mol is considered indicative of meaningful ligand–receptor interaction in molecular docking studies. Therefore, this comparative analysis reinforces the validity of our docking approach and supports the potential of Hyp-GAR and GA-Hyp-GAR as potent ACE inhibitors.

### 2.4. Inhibition Patterns of Hyp-GAR and GA-Hyp-GAR

**Figure 4:**
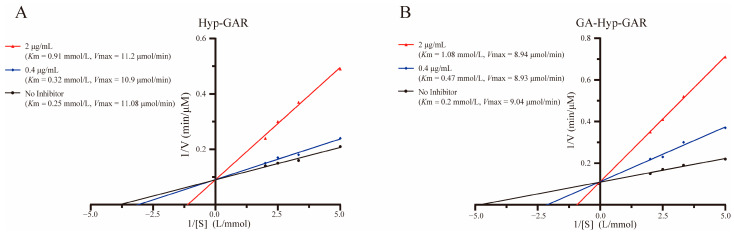
Lineweaver–Burk plots illustrating the inhibitory effect of peptides Hyp-GAR (**A**) and GA-Hyp-GAR (**B**) on angiotensin I-converting enzyme activity.

Lineweaver–Burk plots (Figure 4A,B) demonstrated that both Hyp-GAR and GA-Hyp-GAR inhibit ACE through a competitive mechanism. In both cases, the regression lines at different inhibitor concentrations intersected near the *y*-axis, indicating that *V*max remained unchanged while *K*m increased with rising inhibitor concentrations—hallmarks of competitive inhibition.

To further support this, apparent kinetic parameters (*K*m and *V*max) were calculated from the Lineweaver–Burk regression equations. For example, the *K*m value of GA-Hyp-GAR increased from 0.20 mmol/L (no inhibitor) to 0.47 and 1.08 mmol/L at 0.4 and 2 μg/mL, respectively, while *V*max remained nearly constant (9.04 vs. 8.94 μmol/min). Similar trends were observed for Hyp-GAR. These results confirm that both peptides inhibit ACE by reversibly competing with the substrate for the active site, consistent with the binding interactions predicted by molecular docking.

### 2.5. Antihypertensive Effect of GA-Hyp-GAR in Spontaneously Hypertensive Rats and Its Impact on AngII, BK, eNOS, and NO

**Figure 5:**
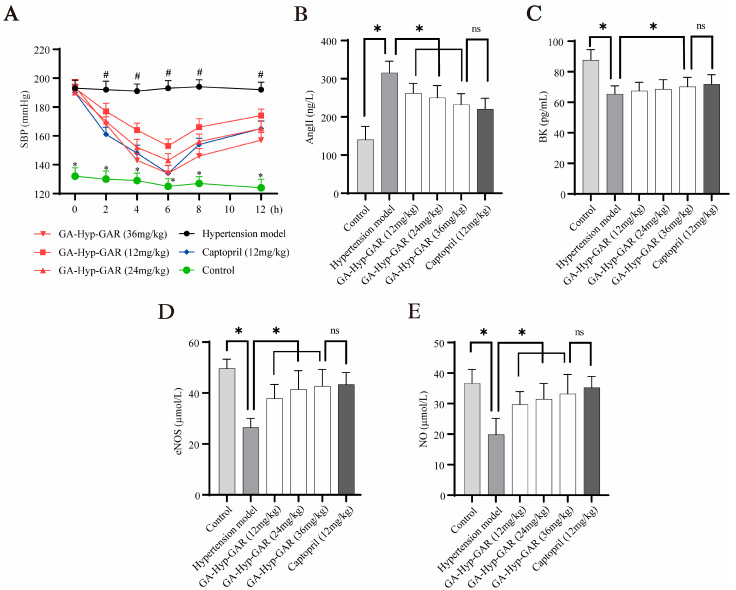
Antihypertensive effects of GA-Hyp-GAR in spontaneously hypertensive rats and modulation of vascular biomarkers. (**A**) Time course of systolic blood pressure (SBP) following oral administration of GA-Hyp-GAR at 12, 24, and 36 mg/kg, compared to captopril (12 mg/kg), the hypertensive model group, and normotensive controls. The maximal SBP reduction was observed at 6 h post-treatment in the high-dose group (60 mmHg), comparable to that of captopril (56 mmHg). (**B**) Plasma levels of angiotensin II (Ang II), which were elevated in the model group, were significantly reduced by GA-Hyp-GAR in a dose-dependent manner. (**C**) Bradykinin (BK) levels, suppressed in hypertensive rats, were restored following GA-Hyp-GAR treatment. (**D**,**E**) GA-Hyp-GAR administration upregulated endothelial nitric oxide synthase (eNOS) and increased nitric oxide (NO) levels compared to the model group. Data are presented as mean ± SD (*n* = 6). * *p* < 0.05 vs. model group; # *p* < 0.05 vs. control group; ns = not significant.

Given its potent ACE-inhibitory activity in vitro, GA-Hyp-GAR was selected for in vivo evaluation of antihypertensive efficacy using SHRs. As shown in Figure 5A, baseline SBP in SHRs averaged 193–194 mmHg, significantly higher than that of normotensive WKY rats (approximately 132 mmHg). Oral administration of GA-Hyp-GAR at doses of 12, 24, and 36 mg/kg resulted in a dose-dependent reduction in SBP, with the maximum effect observed at 6 h post-administration. In the high-dose group (36 mg/kg), SBP decreased from 194 mmHg to 134 mmHg, representing a 60 mmHg (30.9%) reduction. This effect was comparable to that of captopril (12 mg/kg), which lowered SBP by 56 mmHg (29.5%) over the same period. The medium- and low-dose GA-Hyp-GAR groups showed reductions of 47 mmHg and 40 mmHg, respectively. All three GA-Hyp-GAR-treated groups exhibited statistically significant reductions in SBP compared to the untreated hypertensive group (*p* < 0.05). Notably, the antihypertensive effect of GA-Hyp-GAR persisted for up to 12 h, indicating sustained bioactivity and supporting its potential as a natural alternative to conventional ACE inhibitors. Despite some interindividual variability observed—particularly at later time points—the blood pressure-lowering effects of GA-Hyp-GAR remained statistically significant compared to the hypertensive model group (*p* < 0.05), and the variability was within the expected range for SHR models commonly used in antihypertensive research.

In addition to blood pressure regulation, the effects of GA-Hyp-GAR on key vasoactive biomarkers were investigated. Serum levels of AngII were significantly elevated in the hypertensive model group (Figure 5B), reaching 224.62% of the levels in the normotensive control group. GA-Hyp-GAR administration led to a significant, dose-dependent reduction in AngII levels. In the high-dose group, AngII concentrations were statistically comparable to those observed in the captopril group (232.01 ± 28.91 pg/mL vs. 220.16 ± 31.67 pg/mL, *p* > 0.05). Conversely, as shown in Figure 5C, plasma BK levels in the hypertensive model group were significantly lower than those in the normotensive control group (61.11 ± 5.07 pg/mL vs. 92.45 ± 8.94 pg/mL, *p* < 0.05). Treatment with GA-Hyp-GAR resulted in a dose-dependent increase in BK levels. In the high-dose group, BK concentrations rose to levels comparable to those in the captopril-treated group (72.65 ± 5.10 pg/mL vs. 71.81 ± 5.87 pg/mL, *p* > 0.05). Figure 5D and Figure 5E further demonstrate that levels of eNOS and NO were markedly reduced in the hypertensive model group by 53.31% and 54.25%, respectively, compared to the normotensive control. Treatment with GA-Hyp-GAR, as well as with captopril, significantly restored both eNOS and NO levels. In the high-dose group, eNOS and NO concentrations were restored to 86.05% and 90.58% of control levels, respectively, with no significant difference observed relative to captopril-treated rats.

### 2.6. Long-Term Dosing Safety Evaluation

**Figure 6:**
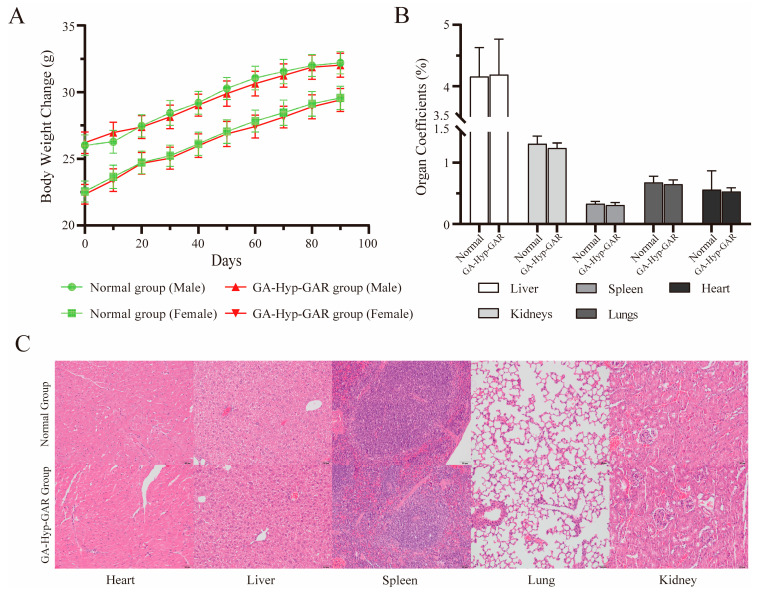
Evaluation of long-term oral safety of GA-Hyp-GAR peptide (50 mg/kg/day) in BALB/c mice over a 90-day period. (**A**) Body weight changes in male and female mice over 90 days. No significant differences were observed between GA-Hyp-GAR-treated and normal groups, indicating normal growth and general health. (**B**) Organ coefficients (organ weight/body weight %) for the liver, spleen, kidneys, lungs, and heart remained within normal physiological ranges in both groups. (**C**) Representative hematoxylin and eosin (H&E)-stained sections of major organs from female mice. No histopathological abnormalities were observed in the GA-Hyp-GAR-treated group, suggesting an absence of tissue toxicity.

To assess the long-term safety of the GA-Hyp-GAR peptide, male and female Balb/c mice were orally administered a daily dose of 50 mg/kg for 90 consecutive days. Throughout the treatment period, no mortality or signs of toxicity were observed. Both sexes maintained stable health, with no behavioral abnormalities, reductions in locomotor activity, or changes in food and water intake compared to the control group. Body weight increased steadily in all groups over the study period (Figure 6A), indicating normal growth and suggesting that the administered dose of GA-Hyp-GAR did not adversely affect the general physiological status. No abnormal changes in body condition, fur quality, posture, or locomotor behavior were observed in either sex. All treated animals exhibited normal grooming behavior and physical activity, and no signs of distress, lethargy, or toxicity were noted. These observations support the absence of systemic adverse effects associated with long-term GA-Hyp-GAR administration at 50 mg/kg/day.

Hematological analysis further supported the safety profile of GA-Hyp-GAR. As shown in Table 1, there were no statistically significant differences (*p* > 0.05) between the treated and control groups in key hematological parameters, including red blood cell count, hemoglobin concentration, red cell distribution width, mean corpuscular hemoglobin, white blood cell count, and platelet-related indices such as platelet count, mean platelet volume, platelet distribution width, plateletcrit, and platelet large cell ratio. These results indicate that long-term exposure to GA-Hyp-GAR does not impair hematopoietic function or induce hematological abnormalities. Serum biochemical analyses likewise revealed no significant differences (*p* > 0.05) between the treated and normal groups in markers of hepatic function—such as aspartate aminotransferase, alanine aminotransferase, and alkaline phosphatase—or renal function, including blood urea nitrogen and creatinine. In the triglyceride analysis, the GA-Hyp-GAR-treated group showed a slight increase in triglyceride levels (2.35 ± 0.48 mmol/L) compared to the control group (1.94 ± 0.40 mmol/L), with a *p*-value of 0.0526. Although this difference did not reach statistical significance (*p* > 0.05), the observed trend suggests a potential increase. However, this change remains within the reported physiological range for BALB/c mice (approximately 0.3–2.5 mmol/L) [22,23]. Other metabolic markers, including total protein, albumin, and glucose, remained within normal physiological ranges and did not differ significantly from the normal group. These findings suggest that GA-Hyp-GAR does not produce hepatotoxic, nephrotoxic, or metabolic side effects under the tested conditions.

To further evaluate potential structural or functional alterations in major organs, mice were euthanized at the end of the study, and the liver, kidneys, spleen, lungs, and heart were excised, weighed, and subjected to histological examination. As shown in Figure 6B, organ coefficients did not differ significantly between the GA-Hyp-GAR-treated and normal groups (*p* > 0.05), indicating the absence of gross organ swelling or atrophy. Histopathological analysis revealed no observable pathological changes in any of the examined organs (Figure 6C). Tissue architecture remained intact, with no evidence of inflammation, necrosis, fibrosis, or other structural abnormalities. These results further confirm the absence of histological toxicity following prolonged oral administration of GA-Hyp-GAR at 50 mg/kg/day.

## 3. Discussion

Both synthetic antihypertensive agents, such as ACE inhibitors (e.g., captopril), and food-derived bioactive peptides have demonstrated that targeting ACE is an effective strategy for hypertension management [24]. In our preliminary experiments, we evaluated various enzymatic hydrolysis strategies for crucian carp swim bladders, employing pepsin, pepsin combined with trypsin, flavourzyme, alkaline protease, and papain. The resulting hydrolysates were assessed for their ACE-inhibitory activities. Notably, hydrolysates generated using pepsin alone or via sequential digestion with pepsin and trypsin exhibited pronounced, dose-dependent ACE-inhibitory effects. In contrast, hydrolysates produced with flavourzyme, alkaline protease, or papain demonstrated relatively low inhibitory activity without a clear dose–response relationship (see Figure S1 provided as Supplementary Electronic Material). Peptides produced by digestion with pepsin and trypsin are also expected to exhibit favorable gastrointestinal stability [25]. These enzymes commonly yield peptides with C-terminal leucine or arginine residues, which are frequently associated with potent ACE-inhibitory activity [26]. Based on these enzymatic characteristics and functional evaluations, pepsin and trypsin were selected as the optimal enzymes for hydrolyzing swim bladder proteins.

RP-HPLC is a commonly used technique for separating bioactive peptides. However, for collagen-derived peptides, the high content of hydrophilic hydroxyproline residues and the C-terminal arginine or lysine residues introduced by trypsin digestion can result in poor retention on conventional C18 columns [27]. This leads to early elution and a potential risk of peptide loss or co-elution with matrix impurities, such as salts introduced during digestion. To address these challenges, we recommend using complementary orthogonal separation strategies, such as size-exclusion chromatography or ion exchange, to improve separation efficiency during peptide purification. In this study, we initially used size-exclusion chromatography to perform a preliminary separation based on molecular weight differences. The low molecular weight fraction (F8) exhibited the most potent ACE-inhibitory activity, consistent with previous studies suggesting that effective ACE-inhibitory peptides typically contain 2 to 12 amino acid residues, with molecular weights ranging from 150 to 800 Da [28,29]. Further purification of the F8 fraction using RP-HPLC identified the R1 subfraction—the most hydrophilic—as having the highest activity.

Peptide identification was performed using LC-MS/MS, a widely used technique that combines high-resolution tandem mass spectrometry with database matching or de novo sequencing for accurate peptide profiling [30]. The predominant peptides identified in the R1 subfraction were Hyp-GAR and GA-Hyp-GAR, with molecular weights of 415 Da and 543 Da, respectively. Both peptides contain polar amino acids, including hydroxyproline (Hyp), glycine (G), and arginine (R), which likely contribute to their hydrophilic nature. These peptides were identified as fragments of collagen, type I, alpha 1a isoform X2 (NCBI RefSeq XP_059407703.1), a key structural protein in connective tissues. Collagen type I plays a critical role in maintaining tissue structure, particularly in the skin, tendons, and bones, suggesting that these peptides may contribute to tissue integrity and elasticity [19]. Chemically synthesized versions of these peptides confirmed their potent ACE-inhibitory activities in vitro.

Previous studies have identified Ala354, His383, Glu384, and Lys511 as key ACE residues involved in binding classical inhibitors like captopril [31]. Consistent with this, molecular docking showed that both Hyp-GAR and GA-Hyp-GAR formed hydrogen bonds or salt bridges with Ala354 and Lys511, suggesting a similar binding mode to captopril. Both peptides also interacted with the catalytic Zn^2^⁺ ion through their glycine residues, potentially disrupting substrate coordination and enhancing their inhibitory effects. The docking results revealed low binding free energies for both peptides, indicating strong binding affinities. Notably, GA-Hyp-GAR showed a lower binding free energy and IC₅₀ value (4.00 μM) compared to Hyp-GAR, suggesting greater potency. Compared to other collagen-derived ACE inhibitors like GPV and AGP [32], which have IC₅₀ values of 10–50 μM, GA-Hyp-GAR exhibited significantly stronger potency. Additionally, the molecular docking results highlighted that GA-Hyp-GAR forms multiple hydrogen bonds with critical residues and coordinates with the Zn^2^⁺ ion, features not present or less pronounced in many other collagen-derived peptides. These interactions likely contribute to its superior binding and inhibitory strength.

Enzyme kinetics further confirmed that both peptides act as competitive ACE inhibitors, as evidenced by increased *K*_m_ values without changes in *V*_max_. In particular, GA-Hyp-GAR showed a steeper slope in the Lineweaver–Burk plot compared to Hyp-GAR, reflecting a more robust inhibitory effect under equivalent conditions. This kinetic behavior aligns with the docking results, in which GA-Hyp-GAR exhibited more extensive interactions within the ACE active site.

Based on its potent ACE-inhibitory activity in vitro, GA-Hyp-GAR was selected for in vivo evaluation using SHRs. ACE plays a central role in blood pressure regulation by converting angiotensin I to angiotensin II, a potent vasoconstrictor [33]. In contrast, bradykinin (BK) promotes vasodilation by stimulating the release of nitric oxide (NO) and endothelium-derived hyperpolarizing factors [34]. However, ACE also degrades BK by cleaving its C-terminal dipeptide, thereby diminishing its hypotensive effect [35]. In a previous study, Cao et al. [14] reported that RGL-(Hyp)-GL, a collagen-derived peptide with an IC₅₀ of 1.44 μM, significantly lowered blood pressure in SHRs at oral doses of 10 and 30 mg/kg. Given the moderately lower potency of GA-Hyp-GAR, we selected oral doses of 12, 24, and 36 mg/kg to ensure sufficient pharmacological exposure while maintaining consistency with prior peptide-based studies. In this study, the oral administration of GA-Hyp-GAR significantly reduced systolic blood pressure in SHRs in a dose-dependent manner, with the high-dose group showing an effect comparable to captopril. Additionally, GA-Hyp-GAR modulated key regulators of vascular tone, including Ang II, BK, eNOS, and NO, suggesting a dual mechanism of action that involves both the renin–angiotensin system and the BK-NO pathway.

Although numerous food-derived ACE-inhibitory peptides have been identified, few studies have systematically assessed their long-term safety [36]. In our toxicity evaluation, mice were orally administered GA-Hyp-GAR at a dose of 50 mg/kg/day (equivalent to 25 mg/kg in rats) for 90 consecutive days. No abnormalities were observed in body weight, food intake, behavioral patterns, SBP, or major organ coefficients. Histopathological examinations revealed no pathological alterations in the heart, liver, spleen, lungs, or kidneys.

## 4. Materials and Methods

### 4.1. Materials and Chemicals

Formic acid (≥99%, LC-MS grade), 4-(2-hydroxyethyl) piperazine-1-ethanesulfonic acid (HEPES, ≥99%, ACS grade), ACE (from rabbit lung, EC 3.4.15.1), acetonitrile (ACN, ≥99%, LC-MS grade), and *N*-[3-(2-Furyl) acryloyl]-Phe-Gly-Gly (FAPGG, ≥99%, ACS grade) were obtained from Sigma-Aldrich Co. (St. Louis, MO, USA). An enzyme-linked immunosorbent assay (ELISA) kit for AngII (catalog no. D731188-0048) was purchased from Sangon Biotech (Shanghai, China). ELISA kits for endothelial nitric oxide synthase (eNOS, catalog no. JL21190-48T) and BK (catalog no. JL21045-48T) were obtained from JONLN Biotechnology Co., Ltd. (Shanghai, China). Nitric Oxide (NO) assay kit (catalog no. A013-2-1) was purchased from Nanjing Jiancheng Bioengineering Institute (Nanjing, Jiangsu, China). All other chemicals were of analytical grade.

### 4.2. Preparation of Crucian Carp Swim Bladder Hydrolysate

The hydrolysate of crucian carp swim bladder (HCSB) was prepared by simulated gastrointestinal digestion as described previously [20]. Fresh swim bladders were rinsed with precooled saline to remove blood and surface lipids, then trimmed into 25 mm × 25 mm pieces, blotted dry, and freeze-dried at −60 °C for 72 h. The dried samples were ground into powder and stored at −80 °C until use. To prepare the hydrolysate, 300 g of freeze-dried powder was suspended in 1.8 L of 4% (*w*/*w*) pepsin solution in 0.1 M KCl-HCl buffer (pH 2.0) and incubated at 37 °C for 2 h with constant shaking. The pH was then adjusted to 8.0 with 1 M NaOH, and trypsin was added (4%, *w*/*w*). The digestion continued for 2 h under the same conditions. Enzyme activity was terminated by heating the mixture at 100 °C for 10 min, then centrifuging at 12,000× *g* for 15 min. The supernatant was collected and lyophilized to obtain the final HCSB powder.

### 4.3. ACE-Inhibitory Activity Assay

ACE-inhibitory activity was measured based on the method reported by Sangsawad et al. [37], with modifications to enhance assay throughput and simplify detection. Instead of using hippuryl-histidyl-leucine and HPLC-based detection of hippuric acid, we employed FAPGG as the substrate, which enables the real-time monitoring of ACE activity through spectrophotometric measurement.

Briefly, 80 µL of the peptide solution was mixed with 20 µL of ACE solution (0.1 U/mL) and 100 µL of 1 mmol/L FAPGG buffer (containing 80 mM HEPES and 0.3 M NaCl, pH 8.3). The mixture was incubated at 37 °C in a microplate reader, and the absorbance at 340 nm was measured every 2 min over a period of 30 min. A control sample was prepared by replacing the peptide solution with 80 µL of 80 mM HEPES buffer.

The rate of FAPGG hydrolysis, determined by the slope of the absorbance decline over a linear interval (10–30 min), was used to assess ACE-inhibitory activity. The percentage inhibition was calculated using the following formula:

ACE-inhibitory rate (%) = [1 − (slope inhibitor/slope control)] × 100(1)

All assays were conducted in triplicate. The IC₅₀ values were determined by fitting a sigmoidal dose–response curve to the data.

### 4.4. Purification of ACE Inhibitor Peptides by Chromatography

HCSB was dissolved in ultrapure water at a concentration of 50 mg/mL, filtered through a 0.22 µm membrane, and subjected to preliminary purification using a Sephadex G-25 column (1.6 × 70 cm, Amersham Bioscience, Uppsala, Sweden). Elution was carried out with ultrapure water at a flow rate of 0.5 mL/min. The absorbance of each eluate was monitored at 280 nm using a UV detector. All fractions were collected and evaluated for their ACE-inhibitory activity. The fraction exhibiting the highest ACE inhibition was lyophilized and further purified by reverse-phase high-performance liquid chromatography (RP-HPLC), equipped with a semi-preparative C18 column and an automatic fraction collector.

Briefly, the lyophilized sample was reconstituted in ultrapure water at a concentration of 20 mg/mL and eluted using a linear gradient of acetonitrile (2–30% over 3–63 min) in 0.2% formic acid at a flow rate of 1.5 mL/min. Elution was monitored at 280 nm, and the resulting peaks were collected and lyophilized for ACE-inhibitory activity assessment. The fraction demonstrating the strongest ACE inhibition was selected for subsequent peptide identification.

### 4.5. Peptide Identification and Synthesis

Peptide sequencing was performed using ultra-high-performance liquid chromatography coupled with linear ion trap-Orbitrap tandem mass spectrometry (UHPLC-LTQ-Orbitrap, Thermo Scientific, Waltham, MA, USA). The lyophilized fraction was reconstituted in ultrapure water at a concentration of 1 mg/mL, and 10 µL of the sample was injected for analysis. Peptide separation was carried out on a C18 column (250 mm × 3 mm, 5 μm particle size; Phenomenex, Torrance, CA, USA). The mobile phase consisted of 0.2% formic acid in water (buffer A) and 100% ACN (buffer B), with the following gradient elution program: 0–2 min, 2% B; 2–19 min, 2–30% B; 19–20 min, 30–95% B; 20–25 min, 95% B; 25–25.5 min, 95–2% B; 25.5–30 min, 2% B. Mass spectrometric data were acquired in data-dependent acquisition (DDA) mode. Each DDA cycle consisted of one full MS^1^ scan acquired in the Orbitrap analyzer at a resolution of 60,000 (*m*/*z* range: 100–1800), followed by five MS^2^ scans of the top five most intense precursor ions, acquired in the Orbitrap at a resolution of 7500. Fragmentation was performed using higher-energy collisional dissociation (HCD) with a normalized collision energy of 40 eV. Ionization was carried out in positive mode with a spray voltage of 3 kV and a capillary temperature of 350 °C. Nitrogen served as both the sheath gas (35 units) and the auxiliary gas (10 units).

Raw data files were processed using PEAKS Studio software (Version: XPro, Bioinformatics Solutions, Waterloo, Canada) for peptide identification [38]. Search parameters were set as follows: digestion enzymes—pepsin and trypsin; parent mass error tolerance—20 ppm; fragment mass error tolerance—0.1 Da; variable modifications—included methionine oxidation and proline hydroxylation. A crucian carp protein sequence database downloaded from NCBI (Carassius carassius—Identical Protein Groups—NCBI) was used as the reference. The identified peptide sequences were synthesized by GL Biochem Co., Ltd. (Shanghai, China), with a purity of ≥98%, and were subsequently used in the analyses.

### 4.6. Molecular Docking

The crystal structure of ACE was retrieved from the RCSB Protein Data Bank (PDB ID: 1O8A, https://www.rcsb.org/structure/1O8A, accessed on 24 March 2024) [39]. Prior to docking, all ligands and solvent molecules were removed. Protein preparation was performed using Chimera v1.13.1 [40], including the correction of non-standard residues, the addition of hydrogen atoms, and the assignment of charges based on the AMBER ff14SB force field [41].

Molecular docking was carried out using AutoDock Vina v1.1.2 [42]. Two peptide ligands, Hyp-Gly-Ala-Arg (Hyp-GAR) and Gly-Ala-Hyp-Gly-Ala-Arg (GA-Hyp-GAR), were individually docked into the active site of ACE, centered at the coordinates (41.586, 37.383, 43.445) within a search space of 35 × 35 × 35 Å^3^. The binding poses with the lowest binding energy were selected as the most favorable conformations for each peptide.

### 4.7. Determination of ACE Inhibition Pattern

The inhibitory mechanisms of peptides Hyp-GAR and GA-Hyp-GAR against ACE were further investigated through kinetic analysis using Lineweaver–Burk plots. Based on the ACE-inhibitory assay described in Section 2.3, reactions were conducted at varying concentrations of the substrate FAPGG (0.2, 0.3, 0.4, and 0.5 mmol/L), in both the absence and the presence of peptide inhibitors at two concentrations (0.4 µg/mL and 2.0 µg/mL). Initial reaction velocities were calculated as previously described, and Lineweaver–Burk double reciprocal plots were constructed by plotting 1/[S] against 1/*v*, where [S] is the substrate concentration and *v* is the reaction velocity. The mode of inhibition for each peptide was determined by analyzing the intersection patterns of the fitted regression lines on the plots [43].

### 4.8. Animal Experiment and Systolic Blood Pressure Measurement

All animal experiments were conducted in accordance with the guidelines of the Animal Ethics Committee of Nanjing Agricultural University, which approved the study protocol (protocol code: NJAU-20240708-23). Captopril and the synthetic peptides were dissolved in physiological saline prior to administration. Male spontaneously hypertensive rats (SHRs; 12 weeks old, 280 ± 15 g, specific pathogen-free) were obtained from Beijing Vital River Laboratory Animal Technology Co., Ltd. (Beijing, China). Animals were housed under standard laboratory conditions (25 ± 2 °C; 12 h light/dark cycle) with free access to standard chow and water. After a one-week acclimatization period, the SHRs were randomly divided into five groups (*n* = 6 per group): (1) a hypertensive model group (received physiological saline), (2) a low-dose GA-Hyp-GAR group (12 mg/kg), (3) a medium-dose GA-Hyp-GAR group (24 mg/kg), (4) a high-dose GA-Hyp-GAR group (36 mg/kg), and (5) a captopril group (12 mg/kg). In addition, normotensive Wistar–Kyoto (WKY) rats (*n* = 6) were included as controls. All treatments were administered once daily by intragastric gavage. In total, 36 rats were used for this experiment. While no formal power analysis was performed, the sample size (*n* = 6) was selected based on standard practices in SHR studies and has been shown to provide sufficient statistical power in similar pharmacological evaluations [44].

Systolic blood pressure (SBP) was measured using a non-invasive blood pressure monitoring system (BP-2000, Softron Co., Tokyo, Japan) at baseline (0 h) and at 2, 4, 6, 8, and 12 h after administration to evaluate the antihypertensive effects of the test peptides, following the method described by Kurita [45]. The levels of AngII, eNOS, and BK were measured using commercial ELISA kits, while NO levels were determined using the Microwell plate method with a specialized assay kit.

### 4.9. Long-Term Dosing Safety Experiment

A total of 40 healthy 7-week-old BALB/c mice (20 males and 20 non-pregnant females), with body weights within ±15% of the cohort mean, were obtained from Yangzhou University. All animals underwent a 7-day acclimation period under specific pathogen-free conditions prior to the experiment. Mice were housed under standard laboratory conditions: ambient temperature of 20–26 °C, relative humidity of 40–70%, and a 12 h light/dark cycle. General appearance, behavior, food intake, and body weight were monitored daily throughout the study [46]. To evaluate the potential subchronic toxicity of the synthetic GA-Hyp-GAR peptide, a 90-day oral administration study was conducted. Mice were randomly divided into two groups (*n* = 10 males and 10 females per group): a normal group and a GA-Hyp-GAR-treated group receiving 50 mg/kg/day via daily intragastric gavage. The normal group received an equivalent volume of ultrapure water via the same route. After 90 consecutive days of treatment, all animals were anesthetized and euthanized 24 h after the final dose. Blood samples were collected via cardiac puncture for hematological and serum biochemical analyses, performed using a Hitachi 3500 fully automated biochemical analyzer [47] (Hitachi High-Tech Science Corp., Tokyo, Japan). Major organs—including the heart, liver, spleen, lungs, and kidneys—were excised, blotted dry, and weighed. Organ coefficients (organ weight/body weight × 100%) were calculated to assess potential physiological or pathological effects associated with long-term GA-Hyp-GAR exposure. Tissues were fixed in 4% paraformaldehyde, embedded in paraffin, sectioned, and stained with hematoxylin and eosin for histopathological evaluation [48]. All animal experiments were conducted in accordance with the ethical guidelines of the Animal Ethics Committee of Nanjing Agricultural University, which approved the study protocol (Protocol No. NJAU-20250120-35).

### 4.10. Statistical Analysis

All data were analyzed using GraphPad Prism version 9.0 (GraphPad Software, San Diego, CA, USA). Results are expressed as the mean ± standard deviation from a minimum of three independent experiments. Statistical comparisons among groups were performed using one-way analysis of variance, followed by Tukey’s post hoc test. A *p*-value of less than 0.05 was considered statistically significant.

## 5. Conclusions and Future Perspectives

In summary, GA-Hyp-GAR is a novel, potent ACE-inhibitory peptide derived from the crucian carp swim bladder, demonstrating strong antihypertensive activity both in vitro and in vivo, with a favorable safety profile in long-term toxicity studies. However, several limitations remain, particularly regarding its gastrointestinal stability, intestinal absorption, and systemic bioavailability, which are critical for oral administration. Future investigations using simulated digestion models, Caco-2 transport assays, and pharmacokinetic analyses are needed to address these factors. Additionally, while our study showed that GA-Hyp-GAR modulates key components of the renin–angiotensin system and the BK-NO pathway, further molecular studies are required to elucidate the exact signaling mechanisms. Future research should also focus on formulation strategies to enhance peptide stability and delivery efficiency. Overall, GA-Hyp-GAR shows strong potential as a natural antihypertensive agent and functional food ingredient, but its translational development will require further pharmacological and mechanistic validation.

## Figures and Tables

**Table 1:** Hematological and serum biochemical parameters in mice treated with GA-Hyp-GAR Peptide (50 mg/kg/day).

Parameter	Normal Group	GA-Hyp-GAR Group	*p*-Value
**Hematological Parameters**			
Red Blood Cells (×10^12^/L)	10.71 ± 0.83	11.13 ± 0.73	0.2451
Red Cell Distribution Width—SD (fL)	32.81 ± 11.68	31.99 ± 7.11	0.8517
Red Cell Distribution Width—CV (fL)	26.58 ± 1.94	25.95 ± 1.49	0.4260
Hemoglobin (g/L)	167.60 ± 13.10	174.70 ± 10.50	0.1978
Mean Corpuscular Hemoglobin (pg)	15.65 ± 0.32	15.70 ± 0.27	0.7101
Mean Corpuscular Hemoglobin Concentration (g/L)	341.80 ± 15.10	355.90 ± 5.50	0.7101
White Blood Cells (×10⁹/L)	4.28 ± 1.72	3.66 ± 1.19	0.3610
Platelet Count (×10⁹/L)	675.50 ± 200.50	683.40 ± 140.00	0.9198
Mean Platelet Volume (fL)	2.05 ± 3.45	2.09 ± 3.52	0.9798
Platelet Distribution Width (fL)	2.91 ± 4.95	3.09 ± 5.00	0.9364
Plateletcrit (%)	0.16 ± 0.27	0.16 ± 0.26	>0.9999
Platelet Large Cell Ratio (%)	3.62 ± 6.29	3.65 ± 6.26	0.9916
**Serum Biochemical Parameters**			
Aspartate Aminotransferase (µkat/L)	2.48 ± 1.03	2.44 ± 0.96	0.9294
Alanine Aminotransferase (nkat/L)	684.30 ± 288.39	701.81 ± 316.56	0.8986
Alkaline Phosphatase (µkat/L)	3.32 ± 0.65	2.99 ± 0.46	0.2065
Blood Urea Nitrogen (mmol/L)	7.81 ± 1.53	8.13 ± 1.33	0.6237
Creatinine (µmol/L)	14.70 ± 5.80	18.13 ± 6.76	0.2390
Total Protein (g/L)	58.21 ± 3.77	58.16 ± 2.09	0.9711
Albumin (g/L)	35.24 ± 1.32	35.60 ± 1.01	0.5021
Total Cholesterol (mmol/L)	2.47 ± 0.23	2.32 ± 0.25	0.1796
Triglycerides (mmol/L)	1.94 ± 0.40	2.35 ± 0.48	0.0526
Glucose (mmol/L)	5.69 ± 3.38	7.79 ± 3.11	0.1654

## Data Availability

The original contributions presented in this study are included in the article/Supplementary Material. Further inquiries can be directed to the corresponding authors.

## Supplementary Materials

The following supporting information can be downloaded at https://www.mdpi.com/article/10.3390/molecules30132812/s1: Figure S1: Inhibition rate (%) of various proteases (Pepsin, Pepsin+Trypsin, Flavourzyme, Alkaline, and Papain) at different concentrations (0.5, 1, and 2 mg/mL).

## Abbreviations

The following abbreviations are used in this manuscript:

ACE	Angiotensin I-converting enzyme
AngII	Angiotensin II
BK	Bradykinin
HCSB	Hydrolysate of crucian carp swim bladder
HEPES	4-(2-hydroxyethyl) piperazine-1-ethanesulfonic acid
FAPGG	*N*-[3-(2-Furyl) acryloyl]-Phe-Gly-Gly
ELISA	Enzyme-linked immunosorbent assay
eNOS	Endothelial nitric oxide synthase
NO	Nitric oxide
RP-HPLC	Reverse-phase high-performance liquid chromatography
UHPLC-LTQ-Orbitrap	Ultra-high-performance liquid chromatography coupled with linear ion trap-Orbitrap tandem mass spectrometry
Hyp-GAR	Hydroxyproline-Gly-Ala-Arg
GA-Hyp-GAR	Gly-Ala-hydroxyproline-Gly-Ala-Arg
SHRs	Spontaneously hypertensive rats
WKY	Wistar-Kyoto
SBP	Systolic blood pressure
LC-MS/MS	Liquid chromatography–tandem mass spectrometry
*V*_max_	Maximum reaction velocity
*K*_m_	Michaelis constant
