# Supplementary material for: Crucian Carp-Derived ACE-Inhibitory Peptides with In Vivo Antihypertensive Activity: Insights into Bioactivity, Mechanism, and Safety
Source: Molecules. 2025 Jun 30;30(13):2812. doi: 10.3390/molecules30132812 (PMC12251161; doi:10.3390/molecules30132812)
Supplement: Supplementary file 1 [file molecules-30-02812-s001.zip › molecules-3721021-supplementary.pdf]

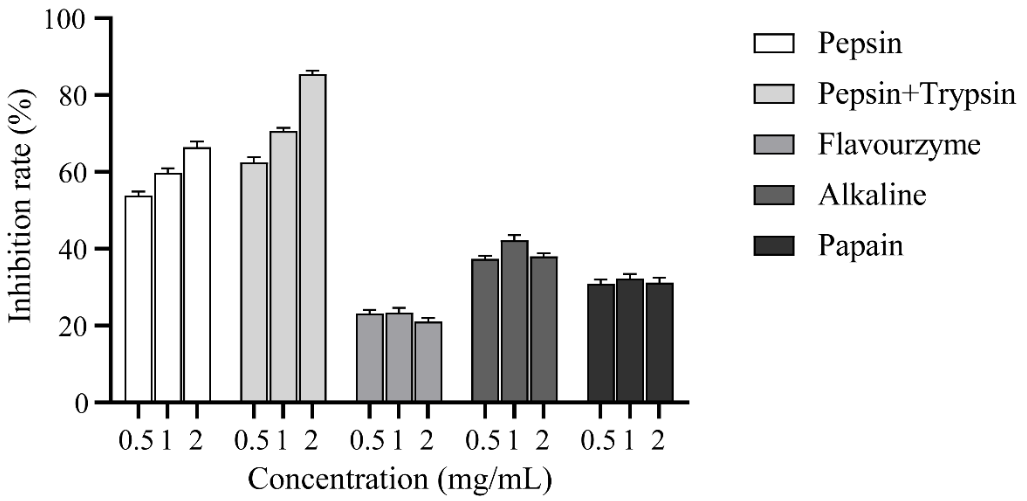

Figure S1: Inhibition rate (%) of various proteases (Pepsin, Pepsin+Trypsin, Flavourzyme, Alkaline, and Papain) at different concentrations (0.5, 1, and 2 mg/mL).
